# Leaky Gut Syndrome Along With Clostridium perfringens Bacteremia in a Neurodegenerative Disease Patient: A Case Report

**DOI:** 10.7759/cureus.75290

**Published:** 2024-12-07

**Authors:** Daisuke Usuda, Manabu Sugita, Pingcheng Shen, Tadashi Umehara, Takeshi Kitamoto

**Affiliations:** 1 Department of Emergency and Critical Care Medicine, Juntendo University Nerima Hospital, Nerima, JPN; 2 Department of Internal Medicine, Hasegawa Hospital, Mitaka, JPN

**Keywords:** anaerobic gram-positive bacteria, bacteremia, c. perfringens, etiology, leaky gut syndrome, neurodegenerative disease

## Abstract

Leaky gut syndrome (LGS) is caused by intestinal epithelial injury and increased intestinal permeability due to a variety of factors, including chronic stress, inflammatory bowel disease, diabetes, surgery, and chemotherapy, resulting in an increased influx of matter from the intestinal lumen causing constipation and bacteremia.

To our knowledge, this is the first known case of LGS along with *Clostridium perfringens* (*C. perfringens*) bacteremia in a neurodegenerative disease patient. The patient was an 81-year-old male with a history of Alzheimer’s disease, cerebral infarction, and diverticulitis in a psychiatric hospital, fed via a nasogastric tube. During hospitalization, he developed a 37.4℃ temperature and disturbance of consciousness evaluated as 3 points on the Glasgow Coma Scale. A follow-up blood examination revealed a white blood cell count of 29,000/µL and a C-reactive protein value of 11.2 mg/dL. Computed tomography revealed an increased concentration of peripheral adipose tissue from the sigmoid colon to the rectum and significant quantities of stool in the rectum. Treatment was initiated with doripenem (DRPM) for sepsis of unknown focus.

*C. perfringens* was subsequently identified in both two blood culture tests. He improved with decreased inflammatory response; DRPM was terminated after 14 days. He remains free of recurrence. We speculate that the LGS in this case may have developed from dopaminergic neuronal decrease and impaired amino acid metabolism caused by chronic hypo-inflammation due to neurodegenerative disease (Alzheimer’s disease). We report the first known case of LGS along with *C. perfringens* bacteremia in a neurodegenerative disease patient.

## Introduction

There is a complex interplay to be found between the immune system and gut microbiota at the gut mucosa level: any disequilibrium between the two can ultimately alter gut permeability, leading to a syndrome known as leaky gut (LG) [1-3]. Further increases in inflammation can be caused by subsequent activation of various inflammatory pathways and alterations to the composition of the gut microbiota with pro-inflammatory bacteria proliferating [2]. Both LG (an intestinal surface barrier defect) and gut dysbiosis (an intestinal microbial population change) are considered to be intrinsic to sepsis [4]. On its own, sepsis can cause dysbiosis, while dysbiosis can exacerbate sepsis [4].

The term “leaky gut syndrome” (LGS) refers to a state of increased intestinal permeability caused by tight junction (TJ) disruption, namely, that of the epithelial barrier permitting translocation of microbial molecules, including Gram-negative bacteria, into circulating blood from the gut; this leads to greater matter influx from the intestinal lumen, in turn causing constipation and bacteremia [4,5]. There is a consistent relationship between microbial translocation and inflammation, as well as a relationship to relatively poor physical function in older patients who have comorbidities [6].

To date, there have been no reports of LGS along with anaerobic Gram-positive bacteria, *Clostridium perfringens* (*C. perfringens*) bacteremia in a neurodegenerative disease patient; therefore, we report such a case, together with a brief review of the literature.

## Case presentation

The patient was an 81-year-old male. He developed a temperature of 37.4°Cand disturbance of consciousness, evaluated as 3 points on the Glasgow Coma Scale, during hospitalization. The patient’s medical history included Alzheimer’s disease (AD), cerebral infarction, and diverticulitis. The patient smoked 20 cigarettes per day, with a 55-year history of smoking, and drank 500 mL of beer per day, with a 55-year history of alcohol consumption. He did not undergo regular medical check-ups. He worked at a printing company until he retired at the age of 65, and had no known allergies. He had been hospitalized in a psychiatric hospital, due to irritability and resistance to care, since he was 79 years old. He was consistently bedridden and required assistance for everyday life activities. Additionally, the patient had no family history of hereditary diseases or malignant diseases.

The patient was 161 cm tall and weighed 51 kg (body mass index: 19.7). At the onset of fever, his vital signs were abnormal: his blood pressure was 76/43 mmHg, his heart rate was 83 regular beats/min, his body temperature was 37.4°C, his oxygen saturation was 82% under ambient air, his respiratory rate was 24/min, and his Glasgow Coma Scale score was 3 points (E1V1M1). Upon physical examination, nothing abnormal was detected, including skin and neurological findings. A routine laboratory examination, taken upon fever, revealed increased values for white blood cells, alkaline phosphatase, gamma-glutamyl transpeptidase, C-reactive protein, activated partial thromboplastin time, and D-dimer, and decreased values for total bilirubin, albumin, and creatine kinase. On the other hand, other values, including complete blood count and biochemistry, were normal (Table 1).

**Table 1:** A routine laboratory examination taken upon fever during hospitalization Neu: Neutrophil, Lym: Lymphocyte, Mon: Monocyte, Eos: Eosinophil, Bas: Basophil

Parameter (Unit)	Measured Value	Normal Value
White blood cells (10^3^/µL)	9.9	3.9–9.7
Neu (%)	65	37–72
Lym (%)	28	25–48
Mon (%)	4	2–12
Eos (%)	2	1–9
Bas (%)	0	0–2
Hemoglobin (g/dL)	10.2	13.4-17.1
Platelets (10^3^/µL)	381	153–346
Aspartate transaminase (IU/L)	17	5–37
Alanine aminotransferase (IU/L)	26	6–43
Lactic acid dehydrogenase (U/L)	173	124–222
Alkaline phosphatase (U/L)	136	38–113
Gamma-glutamyl transpeptidase (IU/L)	90	0–75
Total bilirubin (mg/dL)	0.29	0.4–1.2
Total protein (g/dL)	6.5	6.5–8.5
Albumin (g/dL)	2.7	3.8–5.2
Creatine kinase (U/L)	30	57–240
Blood urea nitrogen (mg/dL)	18	9–21
Creatinine (mg/dL)	0.82	0.6–1
Amylase (IU/L)	110	43–124
Sodium (mEq/L)	140	135–145
Potassium (mEq/L)	4.6	3.5–5
Chloride (mEq/L)	104	96–107
C-reactive protein (mg/dL)	3.5	0–0.3
Procalcitonin (ng/mL)	0.2	0-0.5
Plasma glucose (mg/dL)	102	65–109
Glycated hemoglobin (NGSP) (%)	5.1	4.6–6.2
Activated partial thromboplastin time (seconds)	42.6	23–36
Prothrombin time (International normalized ratio)	1.12	0.85–1.15
D-dimer (μg/mL)	8.1	0–1

**Figure 1:**
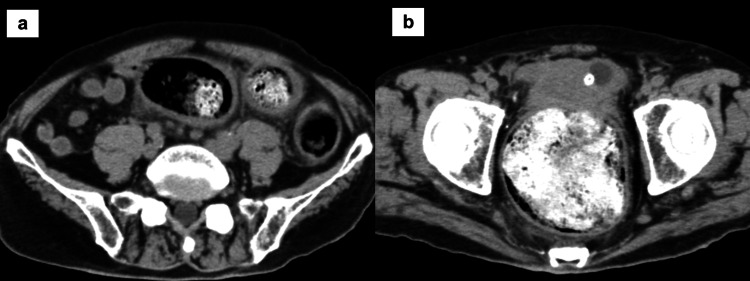
Abdominal computed tomography scan taken upon fever during hospitalization a) Sigmoid colon level. An increased concentration of peripheral adipose tissue is visible in the sigmoid colon. b) Rectum level. A significant amount of stool is confirmed in the rectum.

A computed tomography scan revealed an increased concentration of peripheral adipose tissue from the sigmoid colon to the rectum, and a significant amount of stool in the rectum (Figure 1a and Figure 1b).

**Figure 2:**
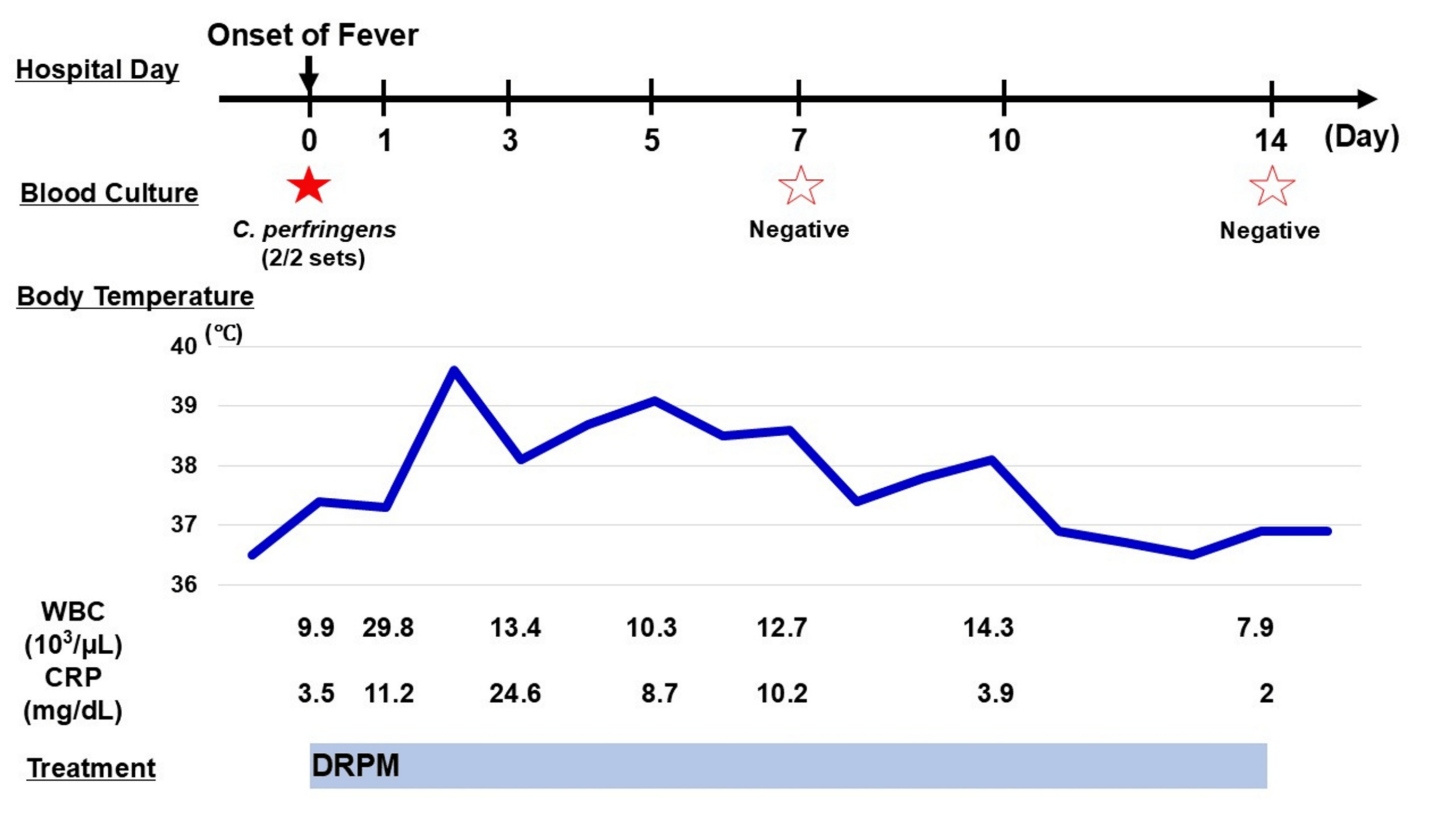
Clinical course of the patient *C. perfringens*: *Clostridium perfringens*; WBC: white blood cell; CRP: C-reactive protein; DRPM: doripenem.

At this point, we established a diagnosis of sepsis of unknown focus. We considered the differential diagnosis as acute infectious gastroenteritis, irritable bowel syndrome, peritonitis, non-occlusive mesenteric ischemia, ischemic enteritis, and peritoneal abscess. Treatment was initiated with doripenem (DRPM). *C. perfringens* was subsequently identified in both two blood culture tests taken upon fever. We continued DRPM based on the results of the drug sensitivity test. The patient improved with a decreased inflammatory response, and DRPM was terminated after 14 days. He remains free of recurrence. The clinical course of the patient is shown in Figure 2. It was speculated that the LGS may have developed from the dopaminergic neuronal decrease and impaired amino acid metabolism caused by chronic hypo-inflammation due to neurodegenerative disease.

## Discussion

We present the first case of LGS along with anaerobic Gram-positive bacteria, *C. perfringens* bacteremia in a neurodegenerative disease patient. Consequently, there is value in reporting this event.

Because the intestinal barrier is an essential element of homeostasis maintenance along the gastrointestinal tract, intestinal hyperpermeability followed by the development of gastrointestinal and non-gastrointestinal diseases can come as a result of lost integrity due to bacterial composition changes, decreased expression levels of TJ proteins, and/or increased pro-inflammatory cytokine concentration [3]. Intestinal hyperpermeability, with increased translocation of Gram-negative bacteria in the gut-brain axis, can enable harmful agents to enter through intestinal epithelial junctions, and then pass into the bloodstream, going on to affect various organs and systems on systemic immune-inflammatory processes [4,5,7]. On the other hand, *C. perfringens* is an anaerobic Gram-positive bacteria, ordinarily found in human intestinal and genital microbiomes, as well as in soil [8].

The pathophysiology of *C. perfringens* bacteremia, especially in the context of gut permeability (intestinal hyperpermeability) with the translocation seems to be different from Gram-negative one. Their strains are characterized by the production of major toxins (alpha, beta, epsilon, and iota), many of these toxins have been demonstrated to contribute to the virulence of bacteria and to play a key role in the pathogenesis of animal infections [9]. In addition, *C. perfringens* enterotoxin (CPE) uses TJ proteins directly as cell surface receptors to attach [9]. CPE, a cytotoxic, pore-forming toxin, uses the claudin family as cellular receptors and it has been shown that it attaches to claudin-3 and claudin-4 of MDCK cell monolayers [9]. Similarly, CPE can bind to specific claudins, resulting in the disintegration of TJs and an increase in the paracellular permeability across epithelial cell layers [9].

After binding, CPE damages the membrane permeability and leads to calcium influx into the cell, resulting in cell damage [9]. In rats, it was shown that the phospholipase C activity of the alpha toxin impaired the intestinal mucosal barrier and increased the permeability of the intestine through the activation of phospholipase [9]. In chickens, it was reported that mucosal addition of *C. perfringens* alpha toxin can impair the intestinal mucosal barrier [9]. Finally, it was observed that the paracellular permeability was higher in tissues from chickens infected with *C. perfringens* [9]. Consequently, as described above, this bacterium could be a causal pathogen of bloodstream infection through impaired intestinal mucosal barrier and increased permeability of the intestine.

In turn, dysbiosis of the gut and impaired permeability of the intestine can alter the gut bacterial metabolite signaling profile, from the gut to the brain [10]. Intestinal permeability risk increases when coupled with a multiple-disease state, or otherwise combined with additional environmental risk factors [7]. The strongest altered intestinal integrity risk factors identified were elevated proinflammatory marker levels, dyslipidemia, hyperglycemia, insulin resistance, anthropometric measurements resembling obesity, advanced disease severity, comorbidity, and consumption of Western-style diets rich in fats and refined carbohydrates [5,7].

Persistent low-grade immune-inflammatory processes, oxidative and nitrosative stress, and hypothalamic-pituitary-adrenal axis activation in the human brain are considered integral elements of increased intestinal permeability pathophysiology [11]. Gut-brain axis abnormalities are additionally associated with psychiatric illness: increasing epithelial permeability leading to LGS can be caused by mood disorders, schizophrenia, alcohol dependence, anxiety disorders, neurodegenerative and neurodevelopmental disorders such as AD or Parkinson's disease, ischemic stroke, chronic inflammatory diseases such as inflammatory bowel disease (IBD), irritable bowel syndrome, gastrointestinal disorders in endurance sports, extra-intestinal diseases including heart diseases, food intolerance, autoimmune diseases such as type 1 diabetes, rheumatoid arthritis, multiple sclerosis, celiac disease, allergies such as asthma, migraines, chronic fatigue syndrome, infections such as upper respiratory infections or human immunodeficiency virus, obesity, type 2 diabetes, unbalanced diet, excessive alcohol consumption, use of non-steroidal anti-inflammatory drugs, and surfactant destroyers such as bile acids [1,10-14].

Of these, excessive alcohol consumption resulting in LGS has been attributed to alcohol-induced liver injury due to bacterial endotoxin portal translocation [13]. In our case, the patient suffered from AD, which is associated with impaired cognition and cerebral accumulation of amyloid-β peptides [1]. The origins of AD involve genetic mutations, epigenetic changes, exposure to neurotoxins, and dysregulation of gut microbiota [12]. The dynamic composition of the gut microbiota, and its metabolites, influence the integrity of the intestinal and blood-brain barriers, contributing to the development of AD [12]. In a recently published study, researchers described interactions between brain protein misfolding and microbiota [1]. More specifically, inflammatory cytokines and neurotoxic molecules that cause neuroinflammation are produced through the activation of microglia and peripheral monocytes that cross the blood-brain barrier [1,12].

This phenomenon has also been described as an abundance of functional bacterial amyloids: amyloid is used by bacteria such as *Proteobacteria*, *Bacteroidetes*, *Chloroflexi*, *Actinobacteria*, and *Firmicutes*, serving as a structural and adhesive material, as a toxin, and as a protection against the host’s innate immunological defenses [1]. The human innate immune system uses several pathways to recognize bacterial amyloid proteins: these involve toll-like receptor 1/2, Nod-like receptor-3 protein, nuclear factor kappa-B, cluster of differentiation 14, and inducible nitric oxide synthase [1]. Further misfolded proteins produced by bacteria could cause a predisposition to tissue damage and to the production of the proinflammatory cytokines that are associated with the onset of dementia [1]. However, further studies will be necessary to more fully characterize AD patients’ gut microbiota composition [1].

As new knowledge, the activation of adenosine A2B receptors in the central nervous system can improve intestinal barrier function through the vagal pathway, and the receptors for adenosine A2B may mediate ghrelin-induced LG improvement in a vagal-dependent way [14]. These findings could help lead to an understanding of the pathophysiology of not only gastrointestinal diseases but also non-gastrointestinal diseases that are associated with altered intestinal permeability [14].

LGS risk factors warrant the attention of clinicians and other healthcare providers to aid in the identification of patients who could be at potential risk of altered intestinal permeability [7]. Unfortunately, there remain no established criteria for measuring intestinal permeability, nor any established gold standard for treatment [15]. Classical methods can be used to assess disruption of the intestinal barrier, which leads to LGS: these include determining the urine concentration of orally administered tracer molecules, or using biomarkers such as lipopolysaccharides (LPS), LPS binding protein, (1-3)-β-D-glucan (major components of gut microbiota), or zonulin in blood plasma [4]. In particular, the grade of LGS correlates with, and can be measured in stool samples through, the presence of zonulin, which is a protein ordinarily found on the intestinal epithelium surface, and which modulates tight junction disruption, to regulate epithelial functions of the intestine [16]. Accordingly, LPS, LPS-binding protein, (1-3)-β-D-glucan, or zonulin in blood plasma, as well as zonulin in the stool could be biomarkers in diagnosing LGS. Furthermore, in in vivo experiments, lactulose, mannitol, or sucralose were optimal probes for the measurement of small intestinal and colonic permeability, therefore, they also could be biomarkers [15].

At present, there are no extant medical guidelines for treating or preventing bacterial translocation in LGS patients; however, a number of studies have suggested that dietary intervention could serve to improve barrier function and restrict invasion of bacteria [3]. In treatment, a priority of any form of intensive care therapy must be the maintenance and/or restoration of intestinal function [17]. The most important measure to take is early enteral nutrition, followed by motility preservation and intestinal microbiome modulation [17]. It is key to reducing intra-abdominal hypertension through individually adapted infusion therapy, patient positioning, drug administration (abdominal compliance), and decompression (using tubes either endoscopically or, in particularly severe cases, surgically) [17].

At present, novel treatments being investigated as new therapeutic approaches to problems in this realm include nutritional regulations, prebiotics, probiotics, fecal microbiota transplantation, bacterial engineering, and vaccination [18]. More specifically, a multi-strain probiotic has been tested that could reduce intestinal permeability in a significant percentage of patients and could serve to improve abdominal pain, stool consistency, and quality of life [19]. Patients who had diabetes or constipation-predominant IBS, known gut-brain interaction disorders with ties to LG, had significantly reduced expression of miR-10b-5p [20]. Researchers performing basic research found that by injecting an miR-10b-5p mimic into mir-10b knockout mice they were able to rescue these molecular alterations and phenotypes [20]. This could lead to new treatment options for millions of patients who suffer from LG-associated conditions [20].

This case study has a limitation: it reviews only a single case report and case series of LGS. Therefore, the actual situation and nature of the disease may differ from the results of the literature review, as a result of reporting bias. Additional studies are needed to further evaluate the impact of clinical presentation, laboratory, microbiology, imaging examinations, and treatment patterns, and the outcomes. In addition, microbiological examinations do not have 100% sensitivity or specificity, meaning that we cannot fully rule out the possibility of the involvement of other organisms not identified through culturing.

## Conclusions

In conclusion, this is, to our knowledge, the first case of LGS with anaerobic Gram-positive bacteria, *C. perfringens* bacteremia, in a neurodegenerative disease patient. The pathophysiology of *C. perfringens* bacteremia, especially in the context of gut permeability with translocation, seems to be different from the Gram-negative one. The incidence of LGS in developed countries, including Japan, is expected to increase as the future brings increasing numbers of patients suffering from dementia.

## References

[1] La Rosa F, Clerici M, Ratto D (2018). The gut-brain axis in Alzheimer's disease and omega-3. A critical overview of clinical trials. Nutrients.

[2] Giambra V, Pagliari D, Rio P (2023). Gut microbiota, inflammatory bowel disease, and cancer: the role of guardians of innate immunity. Cells.

[3] Twardowska A, Makaro A, Binienda A, Fichna J, Salaga M (2022). Preventing bacterial translocation in patients with leaky gut syndrome: nutrition and pharmacological treatment options. Int J Mol Sci.

[4] Chancharoenthana W, Kamolratanakul S, Schultz MJ, Leelahavanichkul A (2023). The leaky gut and the gut microbiome in sepsis - targets in research and treatment. Clin Sci (Lond).

[5] Binienda A, Twardowska A, Makaro A, Salaga M (2020). Dietary carbohydrates and lipids in the pathogenesis of leaky gut syndrome: an overview. Int J Mol Sci.

[6] Kavanagh K, Hsu FC, Davis AT, Kritchevsky SB, Rejeski WJ, Kim S (2019). Biomarkers of leaky gut are related to inflammation and reduced physical function in older adults with cardiometabolic disease and mobility limitations. Geroscience.

[7] Leech B, McIntyre E, Steel A, Sibbritt D (2019). Risk factors associated with intestinal permeability in an adult population: a systematic review. Int J Clin Pract.

[8] Beljan A, Blagec V, Bronic A, Pavić M (2024). Hematological investigations in a case of intravascular hemolysis due to Clostridium perfringens infection. Biochem Med (Zagreb).

[9] Awad WA, Hess C, Hess M (2017). Enteric pathogens and their toxin-induced disruption of the intestinal barrier through alteration of tight junctions in chickens. Toxins (Basel).

[10] Zhang W, Dong XY, Huang R (2023). Gut microbiota in ischemic stroke: role of gut bacteria-derived metabolites. Transl Stroke Res.

[11] Le NP, Altenburger MJ, Lamy E (2023). Development of an inflammation-triggered in vitro "leaky gut" model using caco-2/HT29-MTX-E12 combined with macrophage-like THP-1 cells or primary human-derived macrophages. Int J Mol Sci.

[12] Nohesara S, Abdolmaleky HM, Thiagalingam S, Zhou JR (2024). Gut microbiota defined epigenomes of Alzheimer's and Parkinson's diseases reveal novel targets for therapy. Epigenomics.

[13] Roy N, Nadda N, Kumar H (2022). Pattern recognition receptor CD14 gene polymorphisms in alcohol use disorder patients and its Influence on liver disease susceptibility. Front Immunol.

[14] Ishioh M, Nozu T, Igarashi S (2021). Activation of central adenosine A2B receptors mediate brain ghrelin-induced improvement of intestinal barrier function through the vagus nerve in rats. Exp Neurol.

[15] Khoshbin K, Khanna L, Maselli D (2021). Development and validation of test for "leaky gut" small intestinal and colonic permeability using sugars in healthy adults. Gastroenterology.

[16] Comini L, Pasini E, Porta R, Olivares A, Testa C, Scalvini S, Vitacca M (2023). Dysbiosis and leaky gut in hyper-inflated COPD patients: have smoking and exercise training any role?. Respir Med Res.

[17] Druml W (2018). [Intestinal cross-talk : the gut as motor of multiple organ failure]. Med Klin Intensivmed Notfmed.

[18] Evrensel A (2023). Microbiome-induced autoimmunity and novel therapeutic intervention. Adv Exp Med Biol.

[19] Ait Abdellah S, Gal C, Laterza L (2023). Effect of a multistrain probiotic on leaky gut in patients with diarrhea-predominant irritable bowel syndrome: a pilot study. Dig Dis.

[20] Zogg H, Singh R, Ha SE (2023). miR-10b-5p rescues leaky gut linked with gastrointestinal dysmotility and diabetes. United European Gastroenterol J.

